# Can flunarizine be used routinely as the first option for childhood headache treatment?: Flunarizine and childhood headache

**DOI:** 10.1097/MD.0000000000029265

**Published:** 2022-07-15

**Authors:** Sevgi Çirakli

**Affiliations:** a Division of Pediatric Neurology, Department of Pediatrics, Faculty of Medicine, Ordu University, Ordu, Turkey.

**Keywords:** flunarizine, headache, childhood, treatment

## Abstract

The prevalence of headache in childhood increases due to environmental factors. Various risk factors in children whose playgrounds are restricted outside and therefore remain inactive. So diagnosis and treatment can be challenging. The aim of this study was to evaluate the experience of flunarizine in childhood headache with a focus on efficacy and success.

We conducted a retrospective observational study of 185 pediatric patients at the tertiary pediatric emergency and pediatric neurology unit between May 2018 and May 2020. Patients with headache for >15 days of a month for at least 3 months were included in the study, whether or not receiving treatment. Also, all patients who had an adequate follow-up period were included in the study. All patients were evaluated by history, physical–neurological examination, blood tests, blood pressure, eye examination, and cranial magnetic resonance imaging. All data were evaluated statistically.

Ninety-eight (53%) of 185 cases were female and 87 (47%) were male. Average age was 11.4 years (min–max, 4–17). There was family history in 51.3% of the cases. The most frequent applicants were in the autumn season (43%), when schools were opened. Organic causes were hypertension in 1 case, brain tumor in 1 case, and papilledema due to idiopathic intracranial hypertension in 2 cases. The other cases were asked to make a 1-month pain chart and grading according to the visual analog scale. In this process, it was stated that painkillers could be used if needed. At the end of the first month, these patients were reevaluated. Flunarizine treatment was initiated in 95 patients who had to use painkillers for >4 times and who described ≥6 pain score according to the visual analog scale. The treatment was discontinued due to sleepiness and weakness in 2 patients. At the end of the third month, a 50% reduction in headache was observed in 82 cases (86.3%).

We used flunarizine as the first choice in all patients and we achieved a high rate of treatment success. Flunarizine can be considered as an alternative option for headache management in terms of low side effects, easy accessibility, and compliance with treatment.

## 1. Introduction

Headache is a common complaint in childhood nowadays.^[1]^ Although it used to be less common in children due to lower stress levels and more regular sleep patterns than adults, but the prevalence of headache in childhood increases due to environmental factors, the increase in the time spent in front of the screen instead of exercising, and the increase in school success expectations. Headache has negative features such as decrease in school success, limitation in daily activities, and decrease in quality of life.^[1,2]^ Therefore, it is important to prevent headache attacks.

Its incidence varies according to age.^[3]^ They stated that 10% to 20% of children under the age of 10 years experience headache in their lives.^[4,5]^ Frequency is higher in males before puberty and in females after puberty.^[6]^ Diagnosis and treatment can be challenging due to its variations.^[1]^ In this study, headache cases treated with flunarizine are presented.

## 2. Patients and Methods

Pediatric cases who applied to the pediatric emergency service and pediatric neurology outpatient clinic with headache between May 2018 and May 2020 were evaluated. One hundred eighty-five patients with an adequate follow-up period were included in the study. Patients with headache for >15 days of a month for at least 3 months were included in the study, whether or not receiving treatment. All patients were evaluated by history, physical–neurological examination, blood tests, blood pressure, eye examination and cranial magnetic resonance imaging. Ethics committee approval by the Ordu University (ODU KAEK 2020/19/182) was obtained for the study, and the medical records of the cases were examined. Informed consent was from the patient’s parent.

In the histories, the age of the cases, the duration and frequency of the attacks, the localization of the pain, character, severity, andfamilial characteristics were examined. Patients without an organic cause were first asked to make a 1-month pain chart and grading according to the visual analog scale (VAS).

Flunarizine treatment was initiated in 95 patients who had to use painkillers for >4 times during a month, ≥6 pain according to the VAS and had headaches for >3 months. Patients’ response to treatment was evaluated. All patients were followed up and treated by the same pediatric neurologist. At the end of the third month, the cases were evaluated.

IBM SPSS (SPSS Inc., Chicago, IL) version 21.0 was used for analyzing data. While evaluating the study data, categorical variables were expressed as number (percentage), normally distributed continuous variables as mean ± standard deviation, and nonnormally distributed continuous variables as median and minimum–maximum. Evaluation of normally distribution is made by the Kolmogorov–Smirnov test.

## 3. Results

Ninety-eight (53%) of the cases were female and 87 (47%) were male. The average age was 11.4 years (min–max, 4–17). When the seasonal distribution of the applications was examined, it was observed that it was the highest with 81 cases (43%) in the autumn season when the schools were opened, and the lowest application rate was in the summer with 25 cases (13.5%). There was a history of frequent headache in the family of 95 cases (51.3%; Table 1). It was learned from the stories that 162 (87%) of the patients had used painkillers because of their headaches for >3 months.

**Table 1:** Features of patients.

Features of patients					
Patients (n) (%)	Female (n) (%)	Male (n) (%)	Mean age (min–max)	Season (n) (%)	Family history (n) (%)
185 (100%)	98 (53%)	87 (47%)	11.4 (4–17)	Autumn 81 (43%)	95 (51.3%)

At the time of admission, the pain intensity of all cases was ≥5 points according to VAS. The average VAS score was 7 (min–max, 5–10). Blood tests including vitamin D and B_12_ were checked in all patients, and missing values were replaced. Organic causes were hypertension in 1 case, brain tumor in 1 case, and papilledema due to idiopathic intracranial hypertension in 2 cases. Etiology-oriented treatments were performed in these patients with organic etiology. The pediatric nephrology department evaluated a 14-year-old patient with hypertension, was diagnosed with idiopathic hypertension, and antihypertensive treatment was initiated. A 12-year-old patient diagnosed with a brain tumor underwent neurosurgery, after which chemotherapy was applied. Acetazolamide treatment was initiated in 7-year-old and 11-year-old patients with papilledema due to idiopathic intracranial hypertension. Follow-up of the cases continues by the relevant clinics.

One hundred eighty-one cases without an organic etiology were classified clinically according to the International Classification of Headache Disorders 3 revised criteria (Table 2).^[1,7]^ According to this classification, 68 patients (36.7%) were in the migraine group and 113 patients (61%) were in the tension headache group. All patients were asked to make a 1-month pain chart. At this stage, it was asked to reduce the time spent in front of the screen, such as mobile phone and tablet and pay attention to sleep patterns.

**Table 2:** IHCD-3 revised classification.

ICHD-3 revised classification
**Primary headache**
Migraine
Tension-type headache
Trigeminal autonomic cephalalgias
Other primary headache disorders
**Secondary headache**
Headache attributed to trauma or injury to the head and/or neck
Headache attributed to cranial and/or cervical vascular disorder
Headache attributed to nonvascular intracranial disorder
Headache attributed to a substance or its withdrawal
Headache attributed to infection
Headache attributed to disorder of homeostasis
Headache or facial pain attributed to disorder of the cranium, neck, eyes, ears, nose, sinuses, teeth, mouth, or other facial or cervical structure
Headache attributed to psychiatric disorder
**Painful cranial neuropathies, other facial pain, and other headaches**
Painful lesions of the cranial nerves and other facial pain
Other headache disorders

ICHD-3 = International Classification of Headache Disorders 3 revised.

Treatment with 5 mg/d flunarizine was started in 95 patients who had to use painkillers for >4 times a month and who described ≥6 pain score according to the VAS, regardless of headache classification. Patients with vitamin D and B_12_ deficiency were given a replacement for treatment. However, vitamin D and B_12_ treatment was not added to the treatment. All of the cases were aged ≥8 years. All patients were advised to stay away from headache-triggering situations, from the screen, and to pay attention to their diet and sleep patterns. Two of the patients were discontinued because of flunarizine complaining of too much sleepiness and fatigue.

There were 80 patients whose headaches were reduced by at least 50% in the first-month follow-up of 93 patients who were treated with flunarizine. Flunarizine at a dose of 10 mg/d was given to 2 patients aged 14 and 15 years old and weighed >50 kg, who did not have adequate improvement in their complaints. Both patients had >50% reduction in headache. As a result, successful results were achieved in 82 (86.3%) of 95 cases at the end of the third month.

## 4. Discussion

Chronic headache describes a headache lasting >3 months and is the most common childhood pain syndrome. Its incidence varies according to age groups.^[3]^ It has been observed that headache has become increasingly common in the childhood age group during the last 30 years. The reason for this is thought to be environmental factors and increased awareness of headache. In our series, 185 cases that could be accepted with a high rate were evaluated.

There is an increase in various risk factors in children whose playgrounds are restricted outside and therefore remain inactive. These are in the form of an increase in the rates of obesity and depression as the time spent in front of the screen during the day increases. Due to the highly increased screen time exposure, inactivity, obesity, and depression are interrelated conditions. In addition, situations such as school failure, stress, and high expectations of success from the child are among the major triggering causes of headaches.^[2]^ In our series, it was observed that it was the highest with 81 cases (43%) in the autumn season, when schools were opened and thus the stress level increased. There was family history in 95 cases (51.3%).

Headache is a major problem in the world and we frequently encounter 2 types. These are tension type and migraine type. Tension-type headache is more common than migraine type. Ninety percent of adults state that they have had this experience once in their lives; again, tension-type headache is observed at a much higher rate than migraine in childhood.^[8,9]^ Sixty-one percent of our cases were tension-type headache in accordance with the literature. Migraine type is a headache condition characterized by sensory or sensitivity. Although the pathophysiology of migraine has not been fully explained, there are hypotheses that neurological inflammation and central sensitization may be caused by atypical pain process and cortical hyperexcitability.^[10,11]^ It has been found that migraine is associated with lifestyle habits such as obesity, unhealthy diet, use of technology, weather, and sleep, and some conditions play a triggering role.^[4]^ In our series, 68 cases (36.7%) were evaluated as migraine-type headache.

Although the frequency of headache was higher in the male group before the prepubertal period, it was found more in girls after the pubertal period.^[6]^ This is thought to be caused by estrogen imbalances during the menstrual period. After adolescence, hypertension-type headache frequently arises from high blood pressure.^[12]^ Ninety-eight (53%) of our cases were girls, 87 (47%) were boys, and the mean age was 11.4 years (min–max, 4–17).

Headache may occur in isolation or with accompanying symptoms. While migraine may have aura or no aura, abdominal migraine, cyclic vomiting, and benign paroxysmal torticollis in childhood are among the headache precursors according to the revised International Classification of Headache Disorders 3 revised criteria and are referred to as migraine-related episodic symptoms in the classification.^[7,13]^ In addition, symptoms such as sore throat, fever, weakness, and neck pain may accompany according to etiological reasons.^[1]^ Because important organic pathologies such as intracranial malignancies, hydrocephalus, cerebral thrombosis, intracranial hypertension, and central nervous system infections can be detected among the causes of childhood headaches, the organic etiology of the presenting patients should be excluded quickly.^[14,15]^ In this respect, attention should be paid to life-threatening causes and warning signs.^[1,16]^ In our series, organic causes were detected in the form of hypertension in 1 case, brain tumor in 1 case, and papilledema due to idiopathic intracranial hypertension in 2 cases.

Childhood headaches are necessarily affected by familial problems. Parental disagreement in childhood, chronic illness of the mother or father, mental illness, alcohol problems of divorced parents, mother or father, not having a planned child, neglect in childhood, and abuse have been observed to lead to a significant increase in the frequency of headaches in the future.^[17]^ In a study examining the lifestyles of children followed up with headache, psychological disorders such as technology use, insomnia, depressive symptoms, air change, nutrition, and obesity were found to be associated with children.^[4]^

Nutritionally, cheeses, sausages, ice cream, chocolate, alcohol, monosodium glutamate, high amounts of caffeine intake, aspartame-containing drinks to avoid weight gain, especially in adolescent girls, hunger diets made to stay weak increase headache attacks. Some patients are particularly sensitive to certain foods, and patients observe the triggering agent or agents can cause symptoms, and in this respect, it is recommended to stay away from triggering environments.^[1]^ In addition, it is necessary to take preventive measures to reduce the frequency and duration of headache attacks in order for children to continue their social and educational activities in optimal conditions. In our series, all patients who were followed up and treated were advised to stay away from the conditions that trigger headache and the screen and to pay attention to their diet and sleep patterns.

There are different options for treatment according to the type and severity of headache, but there is still no common consensus. If patients who apply to the emergency department need respiratory or vital support, these situations should be resolved first. In the acute treatment of patients with stable life functions, it is recommended to reduce pain and avoid triggering factors. Especially for photophobia and phonophobia, rest in a dark and quiet environment is recommended. Hydration shows significant benefit. If there is vomiting, antiemetic can be applied in addition to hydration. Acute attack treatment in children can be in the form of administering paracetamol at 10 to 15 mg/kg and ibuprofen at 10 mg/kg.^[18]^ It is stated that patients who mostly applied to the emergency or outpatient clinic received analgesics and did not benefit; in this case, the doses taken may be controlled and additional treatment may be required.^[1,19]^ Then, action should be taken according to etiological reasons. In cases with organic etiology, treatments should be planned according to the cause. Follow-up of 4 cases with organic etiology in our series is continuing by the relevant clinics. There are different alternatives in the treatment of childhood headaches without an organic etiology. Various medications can be given to prevent headache attacks. The choice of pharmacological agents is chosen according to the presence of comorbidity, severity, and frequency of attacks.

Antihypertensives, antidepressants, calcium channel blockers, and anticonvulsants (topiramate, valproic acid) can be used in the treatment.^[20,21]^ The use of melatonin may be beneficial for insomnia problem.^[22–24]^ Alpha agonists, beta-blockers, botulinum toxin-A, calcium channel blockers, antiepileptics, tricyclic antidepressants, and selective serotonin reuptake inhibitors can be used in the treatment of migraine.^[8]^ Triptans (ergot alkaloids) have not been tested in children <18 years of age, and their safety for children and adolescents has not been fully proven. However, there are also triptans approved for use in children. The 2012 American Academy of Neurology guideline recommends beta-blockers, especially propranolol and metoprolol, in the first line of antimigraine treatment.^[25]^ In Europe, it has been observed that beta-blockers, antiepileptics, calcium channel blockers, and tricyclic antidepressants are more prominent.^[8]^

Flunarizine is a long-acting calcium channel blocker and was used in 1970 for occlusive vascular diseases. Its high lipophilic structure crosses the blood–brain barrier and is more concentrated in the tissue than in the blood.^[26,27]^ The half-life of the drug is around 7 to 10 days, and a single dose per day is recommended due to its long half-life.^[28]^ Flunarizine is also used in Sturge–Weber syndrome and hemiplegic migraine. The mechanism of action of flunarizine, which is also used in migraine treatment, is unknown.^[26]^ It is likely that the calcium and dopamine antagonism of flunarizine is thought to act on brain subcortical target cells. Some studies have also shown this positive effect.^[29,30]^ Mohamed et al^[20]^ reported in their study that flunarizine reduced the frequency of attacks by 57%. Again, Sorge et al^[31]^ reported that flunarizine decreased both the frequency of attacks and the duration of the attacks in their study consisting of 70 cases. In a series of 200 cases conducted in England, flunarizine was reported to be effective and safe in treatment.^[26]^ We started flunarizine at a dose of 5 mg/d for all patients and continued for at least 3 months for those who tolerated the treatment. As we did not get enough response from the treatment in our 2 patients, we applied the treatment at 10 mg/d. Normally, it is possible to gradually increase the dose of flunarizine to a maximum of 20 mg/d in cases of ≥50 kg. We administered flunarizine once a day, half an hour before going to bed. Considering the side effects of flunarizine, there are cases that reported mood instability, weight gain, sedation, and seizures, albeit rarely.^[32]^ We had to stop flunarizine due to sedation in only 2 of our patients. We think the reason we saw few side effects is that we started treatment with the lowest dose.

There are certain reasons for using flunarizine as the first choice in treatment. Flunarizine has been used in antimigraine treatment for 3 decades.^[26]^ It has few side effects and blood level monitoring of the drug is not required. Although medical treatment is considered as the first choice for pediatric headache, this may not be at present the most evidence-based approach.^[33]^ Many studies have also shown this positive effect in the literature.^[29,30]^ It is essential to offer adolescents who suffer from headache a timely psychological and medical management in order to implement quality of health and quality of life and prevent headache later in life.^[34]^ In addition, taking a drug that is effective may strengthen the therapeutic alliance between patient and clinician. According to the experience we have gained from our clinical practice, the treatment is continued for at least 3 months, and the treatment is terminated and periodically followed in cases without complaints.

Our study has some limitations. First, our study has been planned retrospectively. So there is no control group for comparing. Second, because of the headache, they have used different drug options in advance of some of their cases, which is unknown. Another missing aspect of the study, regardless of the type of headache, is starting the flunarizine treatment in all cases and cannot be provided a homogeneous group.

## 5. Conclusion

In conclusion, Different treatment options are available according to the type and severity of headache, but there is still no common consensus. Flunarizine shows that it can be considered as an alternative option in a safe and effective manner in headache management because of its low side effects, easy accessibility, and high treatment success.

## Author contributions

All steps: Sevgi Çirakli
